# S1PR1 promotes proliferation and inhibits apoptosis of esophageal squamous cell carcinoma through activating STAT3 pathway

**DOI:** 10.1186/s13046-019-1369-7

**Published:** 2019-08-22

**Authors:** Yan Liu, Yingru Zhi, Haizhu Song, Mingzhu Zong, Jun Yi, Guoxin Mao, Longbang Chen, Guichun Huang

**Affiliations:** 1 Department of Medical Oncology, Jinling Clinical Medical College of Nanjing Medical University, Nanjing, Jiangsu Province, China; 2 Department of Oncology, Affiliated Hospital of Nantong University, Nantong, Jiangsu Province, China; 3 Department of Medical Oncology, Jinling Hospital, Medical School of Nanjing University, Nanjing, Jiangsu Province, China

**Keywords:** ESCC, S1PR1, STAT3, Proliferation, Apoptosis

## Abstract

**Background:**

Esophageal squamous cell carcinoma (ESCC) is one of the most common cancers worldwide, which lacks effective biomarkers for prognosis. Therefore, it is urgent to explore new potential molecular markers to discriminate patients with poorer survival in ESCC.

**Methods:**

Bioinformatics analysis, qRT-PCR, and western blot were applied to investigate S1PR1 expression. CCK-8 assay, colony formation assay, flow cytometry dual staining assay, and immunofluorescence were performed to examine cell proliferation ability and apoptosis rate. Mouse xenograft model of TE-13 cells was established to confirm the roles of S1PR1 in vivo. Gene set enrichment analysis (GSEA) was used to investigate the downstream signaling pathways related to S1PR1 functions. Co-IP was performed to verify the direct binding of S1PR1 and STAT3. Western blot was applied to determine the phosphorylation level of STAT3. Immunohistochemistry was conducted to identify protein expression of S1PR1 and p- STAT3 in tumor tissues.

**Results:**

In the present study, we found that S1PR1 expression was higher in ESCC patients and was a potential biomarker for poor prognosis. Silencing S1PR1 expression inhibited proliferation, and increased apoptosis of ESCC cells, while overexpression of S1PR1 had opposite effects. Mechanistically, S1PR1 played the roles of promoting proliferation and attenuating apoptosis through directly activating p-STAT3. Furthermore, in vivo experiments verified this mechanism.

**Conclusion:**

Our findings indicated that S1PR1 enhanced proliferation and inhibited apoptosis of ESCC cells by activating STAT3 signaling pathway. S1PR1 may serve as a prognostic biomarker for clinical applications.

**Electronic supplementary material:**

The online version of this article (10.1186/s13046-019-1369-7) contains supplementary material, which is available to authorized users.

## Background

Esophageal cancer is one of the most common cancers worldwide with a high mortality rate [1, 2]. Due to different etiology, the pathologic types of esophageal cancer between western countries and China have many differences and esophageal squamous cell carcinoma (ESCC) is the predominant type in China. Despite a steady decline in mortality for esophageal cancer over the past two decades, the 5-year survival rate is still less than 53% in China [3]. The high mortality of ESCC in China might be caused by advanced cancer stage at diagnosis, tumor heterogeneity and insufficient tumor prognostic factors, etc. [4]. Therefore, it is urgent to explore definite molecular markers to discriminate patients with poorer survival, who need intensive treatments.

It was revealed that the increased level of Sphingosine-1-phosphate (S1P) could be activated by Sphingosine kinase (SphK), and it could function as a second messenger intracellularly or be secreted out of cells, then bound to Sphingosine-1-phosphate receptors (S1PRs) [5]. S1PR1, a member of the G-protein-coupled receptors, including S1PR1–5, was predominantly expressed in various cells. Activation of S1PR1 has been reported to be engaged in the regulation of many malignant biological phenotypes of tumors, such as tumor growth, invasion, migration, angiogenesis, and radio-resistance, functioning as an important oncogenic regulator in many cancers [6, 7]. For example, a previous study exhibited that the expression of S1PR1 mRNA was associated with tumor staging in esophageal carcinoma [8]. Moreover, accumulating studies suggested that S1PR1 coupled to Gi and activated downstream signaling pathways, including the PI3K/AKT, PI3K/Rac, Ras/ERK, NF-κB and PLC signaling pathways [9, 10]. However, the roles of S1PR1 in modulating the proliferation and apoptosis of ESCC cells remain to be elucidated.

In this study, through analyzing the TCGA database, we found that the expression of S1PR1 was significantly higher in ESCC patients with poorer prognosis. Furthermore, we analyzed the expression of S1PR1 in ESCC tissues chip using immunohistochemistry (IHC) and investigated the functions of S1PR1 in ESCC proliferation and apoptosis in vitro and in vivo*.*

## Methods

### Patients and clinical samples

ESCC paraffin-embedded tissues of 127 patients were obtained from the Department of Pathology, Affiliated Hospital of Nantong University between Jan, 1st, 2001 and Dec, 31th, 2005. All the patients were followed up to Dec, 31th, 2016. Clinical samples were collected with informed written consent from patients, and the ethical approval was granted by the Review Board of Affiliated Hospital of Nantong University.

### Immunohistochemistry

Four micrometers specimen sections were deparaffinized and rehydrated; then antigen retrieval was performed. After that, endogenous peroxidase activity was blocked. After incubation of primary and secondary antibody, the slides were incubated with diaminobenzidine (DAB) and finally counterstained with hematoxylin. Primary antibodies are listed as follows: Ki-67 (1:500, Proteintech, USA), S1PR1 (1:200, Abcam, USA), p-STAT3 (1100, Abcam, USA), TUNEL staining (Roche, USA). The immunostaining intensity was classed into four categories: negative (value = 0), weak (value = 1), moderate (value = 2) and strong (value = 3). The percentage of positive cell was classed into four categories: 0–25% (value = 0), 26–50% (value = 1), 51–75% (value = 2) and 76–100% (value = 3).

### TCGA data analysis

RNA-Seq data from the TCGA patients’ data set portal (https://cancergenome.nih.gov/) was analyzed for the expression of S1PR1 in esophageal cancer. Information of the ESCC patients including survival times were downloaded from the TCGA esophageal cancer data set portal (TCGA accession codes were listed in Additional file 1: Figure S1A). The selected esophageal cancer patients were assigned into two groups according to patients’ over survival (OS). Gene set enrichment analysis (GSEA)were conducted to compare mRNA expression profiles between the two groups.

### Cell lines and cell culture

Human ESCC cell lines TE-1, TE-13, kyse150, kyse450, ECA-109 and normal epithelial cells HEEC cell lines were cultured in RPMI-1640 medium (GIBCO, USA) supplemented with 10% fetal bovine serum (GIBCO, USA). All the cells were incubated at 37 °C under 5% CO_2_.

### Cell counting kit-8 assay

ESCC cells were seeded onto 96-well plates overnight. CCK-8 (Dojindo Molecular Technologies, Japan) reagent was added to each well (with 1:10 dilution). The cells were incubated for another 2 h, followed by the detection of 450 nm absorbance using a microplate reader (Bio-Rad, Model 680, USA). The experiments were repeated at least three times. IC50 values of ESCC cells were calculated by curve simulation.

### Colony formation assay

Single cell suspensions were seeded into 6-well plates with about 400 cells each well. After 14 days of culture or ocular cell clusters were formed, cells were fixed in 4% formaldehyde and stained with violet crystal (0.1%). The numbers of visible colonies were counted. Each experiment was performed three times.

### Flow cytometry

Apoptosis was measured by Annexin V-fluorescein isothiocyanate (FITC) apoptosis detection kit (Oncogene Research Products, Boston, MA) according to manufacturer’s instruction. For cell cycle assays, cells were fixed in 70% ethanol and stained with PI, followed by flow cytometry analysis. The experiments were performed on the BD FACScan (Becton Dickinson). All of the analysis was performed in triplicate.

### Retroviral construction infection and transfection

Two most effectively plasmid (siRNAs#2, 5′-TCAGCCTCCGTGTTCAGTCTCCTCGCCAT-3′; siRNAs#3, 5′-CCGCTCTACCACAAGCACTATATCCTCTT-3′) for the functional experiments targeting S1PR1 was cloned into piLenti-GFP-pure vector (siRNA). Lentivirus packaging was supported by Genechem (Shanghai, China). Lentivirus vector expressing S1PR1 (LV-S1PR1) and negative control were purchased from Genechem Company (Shanghai, China). ESCC cells were seeded into 6-well plates (2 × 10^5^cells/well) and infected with lentivirus with polybrene (Sigma, USA). Puromycin (1 μg/ml, Sigma, USA) was utilized to screen the stably infected cells.

### Quantitative reverse-transcription polymerase chain reaction (qRT-PCR)

Total RNA was extracted from cells with Trizol reagent (Takara, Japan). Reverse transcription was conducted using the Prime Script RT Reagent Kit (Takara, Japan). Real-time quantitative PCR was performed in a reaction mix of SYBR Green (Takara, Japan) on StepOne Real-Time PCR System (Applied Biosystems). The mRNA level was normalized against β-actin. Sequences of primers used for qRT-PCR in this study were listed in Additional file 1: Table S1.

**Table 1:** Clinico-pathologic characteristics of ESCC patients

Characteristics	Total 127	S1PR1		χ2	
		Low expression(65)	High expression(62)		*P*-value
Age					
< 60	30	18	12	1.223	0.269
≥ 60	97	47	50		
Gender					
Male	80	38	42	1.172	0.279
Female	47	27	20		
Histological grade					
G3	54	19	35	10.963	0.005*
G2	44	25	19		
G1	29	21	8		
T classification					
T1	34	23	11	6.941	0.031*
T2	48	25	23		
T3	45	17	28		
N classification					
Absent (0)	91	55	36	11.013	< 0.01*
Present (1/2/3)	36	10	26		
M classification					
M0	122	63	59	0.26	0.61
M1	5	2	3		
Clinical stage					
I	28	21	7	8.076	0.018*
II	65	31	34		
III/IV	34	13	19		

*,P < 0.05

### Co-immunoprecipitation (co-IP)

Total protein in TE-13 was harvested as input. The supernatants were incubated with 30 μl protein A/G agarose (Santa Cruz Biotechnology, USA) and 6 μl S1PR1 antibody (Abcam, USA) or 6 μl STAT3 antibody (Cell Signaling Technology, USA) at 4 °C overnight. Then, deposited immune complexes were analyzed by Western blotting using antibodies against S1PR1 and STAT3.

### Immunofluorescence assay

Cells were seeded on glass coverslips in 6-well plates fixed in 4% formaldehyde solution and permeabilized with 0.5% Triton X-100/PBS. Cells were blocked with 5% BSA for 30 min at room temperature and incubated with primary antibody against p-STAT3 (1:400, Cell Signaling Technology) at 4 °C overnight, followed by incubation with fluorescent dye-conjugated secondary antibody (Invitrogen, USA) for 2 hrs, and then mounted with DAPI (SouthernBiotech, USA). Finally, images were taken under an inverted fluorescence microscope (Carl Zeiss, Germany).

### Western blot

The cells were lysed, and total proteins were collected. Total protein was separated by SDS-PAGE and transferred onto polyvinylidene fluoride (PVDF) membrane (Millipore, USA). The membrane was then blocked with 5% bovine serum albumin in TBST for 2 h at room temperature and incubated with primary antibodies at 4 °C overnight. Following the incubation with secondary antibodies for 2 h at room temperature, proteins on the membrane were visualized with a chemiluminescence kit (Thermo Scientific, USA). Primary antibodies are listed as follows: PCNA (1:1000, Abcam), STAT3 (1:1000, Cell Signaling Technology), p-STAT3 (1:1000, Cell Signaling Technology), cyclinD1 (1:1000, Cell Signaling Technology), BCL-XL (1:1000, Cell Signaling Technology), cleaved caspase-3 (1:1000, Cell Signaling Technology), GAPDH (1:1000, Abcam).

### Mice xenograft models

BALB/c-Foxn1^nu^/Nju nude mice (male, 6 weeks) were provided by the Department of Comparative Medicine of Model Animal Research Center of Nanjing University. Approximately 1 × 10^7^ TE-13 cells were implanted into the posterior flank of nude mice. Xenograft size and mice body was measured every other day and calculated by using the eq. V (mm^3^) = (length × width^2^)/2. When the tumors grew to 100 mm^3^, DMSO with SH-4-54 was given through intraperitoneal injection at a concentration of 5 mg/kg every 3 days. Body weight was measured every other day. All mice were sacrificed after 23 days, and the tumor tissues were used for subsequent studies.

### Statistical analysis

Statistical analysis was performed with SPSS 23.0 software (SPSS, Chicago, USA). Two-tailed Student’s t-test and Fisher’s exact test were used for comparison of means between two groups. Long-rank test was applied for survival comparison. Multiple group comparisons were analyzed with ANOVA. *P* < 0.05 was considered statistically significant.

## Results

### High expression of S1PR1 in ESCC correlated with poorer survival

**Fig. 1:**
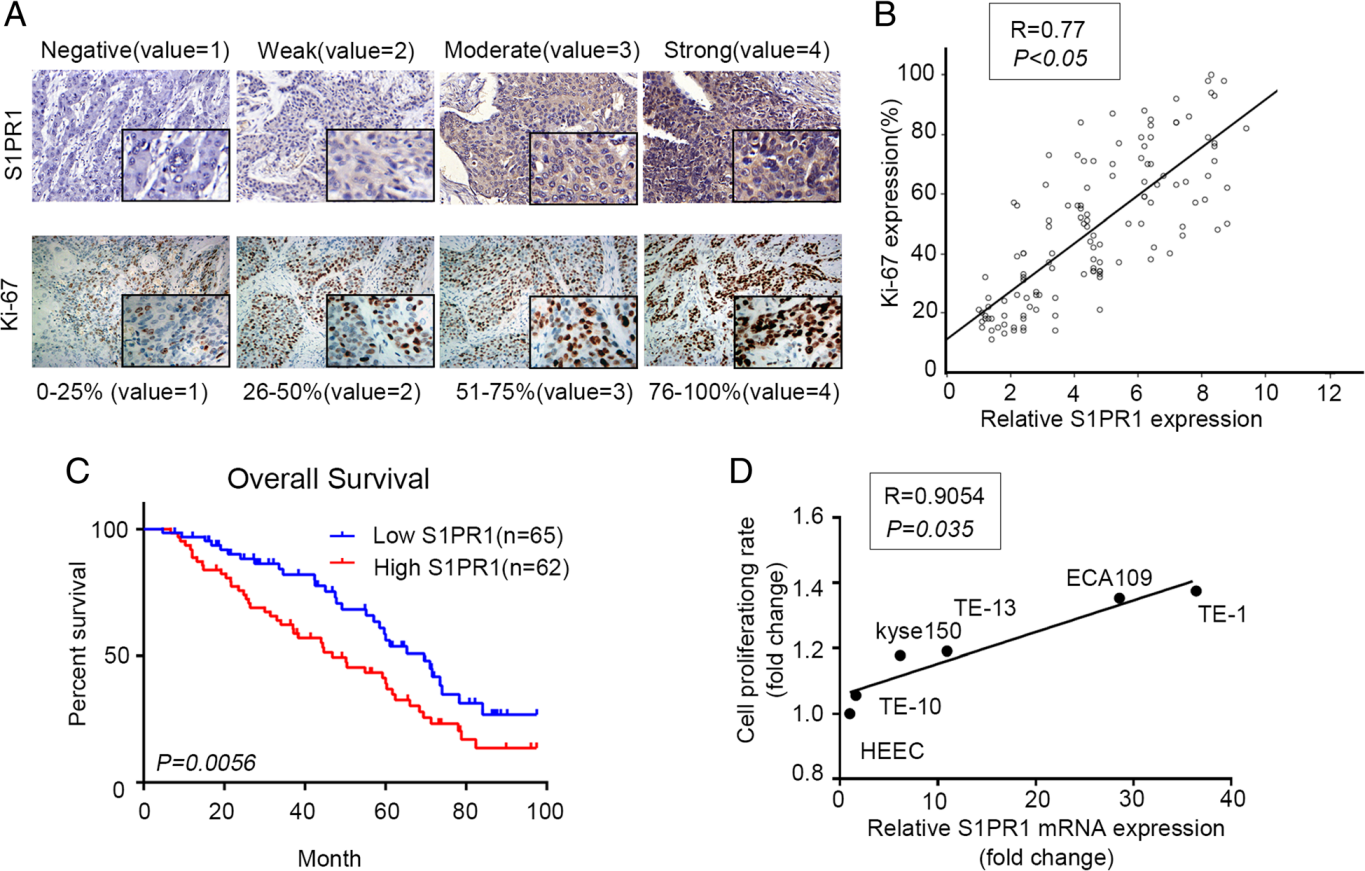
High expression of S1PR1 was correlated with poor prognosis of ESCC patients. a. Representative images from immunohistochemically staining of S1PR1 and Ki-67 in ESCC patient tissues. b. Pearson correlation analysis of the expression of S1PR1 and Ki-67 in ESCC tissues. Statistical significance was determined by Student’s t test. *p < 0.05*. c. The Kaplan-Meier survival curve of overall survival (OS) according to S1PR1 immunostaining in 127 cases of ESCC patients. Patients with high S1PR1 expression have shorter OS than those with low S1PR1 expression (log-rank test, *P < 0.01*). d. Pearson correlation analysis of S1PR1 mRNA expression and cell proliferation ability in ESCC cell lines. Statistical significance was determined by Student’s t test, *p < 0.05*

We first investigated S1PR1 expression in the tissues chip of ESCC. By multiplying the values of immunostaining intensity and positive cell percentage, four expression levels of S1PR1 were designated: negative (Score 0–1), weak (Score 2–4), moderate (Score 5–7) and strong (Score 8–12). The percentage of Ki-67 positive cells was assigned to four categories: level 1(0 to 25%), level 2 (26 to 50%), level 3(51 to 75%) and level 4 (76–100%) (Fig. 1a). High expression of S1PR1 was correlated with high proliferation rate of ESCC cells (Fig. 1b). We next addressed the clinical significance of S1PR1 expression in ESCC by evaluating correlations between S1PR1 expression and clinic-pathological features in the 127 patients. We found that high expression of S1PR1 significantly correlated with high histological grade, lymph node metastasis and advanced clinical stage (Table 1). Additionally, ESCC patients with high S1PR1 expression have a shorter overall survival (median survival 47 months,95%CI 0.9370 to 2.360) comparing to patients with low S1PR1 expression (median survival 70 months, 95%CI 0.4236 to 1.067) (Fig. 1c). We further investigated S1PR1 mRNA expression in the TCGA database. Consistently, S1PR1 expression is significantly higher in the group with poor prognosis (Additional file 1: Figure S1A). Taken together, S1PR1 expression could be a prognostic factor in ESCC patients. Based on the clinical significance of S1PR1, we further investigated its biological role in the proliferation ability of ESCC cells. We detected S1PR1 expression by quantitative real-time PCR and analysis cell proliferation rate in five ESCC cell lines (TE-1, TE-10, kyse150, TE-13, ECA109) and one normal esophageal epithelial cell line (HEEC). As shown in Fig. 1d, the expression of S1PR1 was positively correlated with the cell proliferation of ESCC cell lines.

### Silencing S1PR1 expression inhibited proliferation and increased apoptosis of ESCC cells in vitro

**Fig. 2:**
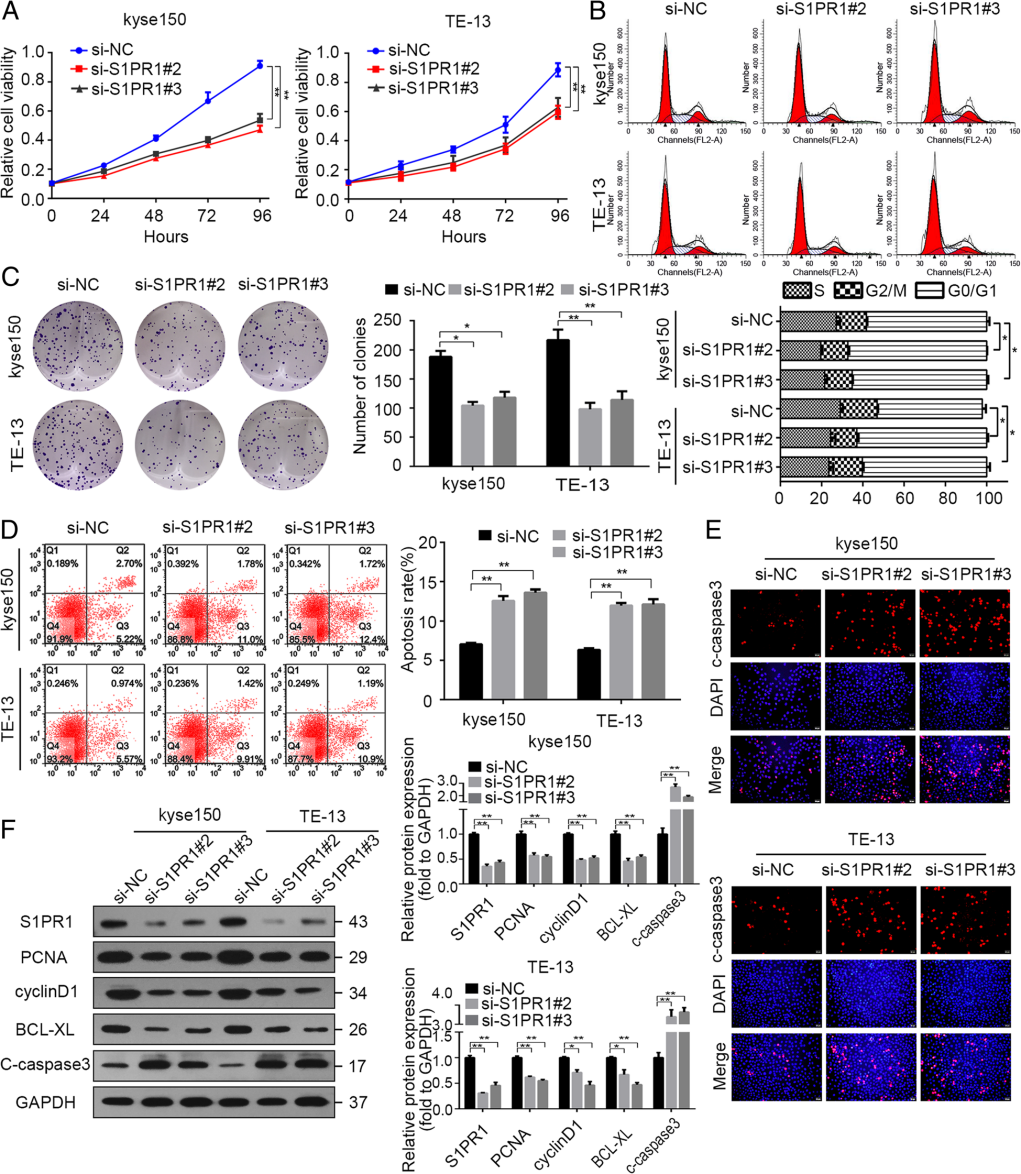
The effects of S1PR1 downregulation on cell proliferation and apoptosis of ESCC cells. a. CCK-8 assay analysis of kyse150 and TE-13 cells transfected with si-NC or si-S1PR1 respectively. b. Flow cytometry analysis of cell cycle distribution proportion in kyse150 and TE-13 cells transfected with si-NC or si-S1PR1 respectively. Representative images and average percentages of cells are shown. c. Colony formation assay of kyse150 and TE-13 cells transfected with si-NC or si-S1PR1 respectively. d. Flow cytometry analysis of apoptotic cells in kyse150 and TE-13 cells transfected with si-NC or si-S1PR1 respectively. The sum of Annexin V positive population and PI positive population is exhibited. e. Immunofluorescent staining of c-caspase3 of kyse150 and TE-13 cells transfected with si-NC or si-S1PR1 respectively. f. Western blot analysis of S1PR1, PCNA, cyclinD1, Bcl-xL, c-caspase3 and GAPDH in kyse150 and TE-13 cells transfected with si-NC or si-S1PR1 respectively

We further selected four cell lines (kyse150, ECA109, TE-1 and TE-13) for the following experiments. To investigate the role of S1PR1 on the proliferation and apoptosis of ESCC cells, S1PR1 expression was knockdown by plasmid-mediated short interfering RNAs (siRNAs) in kyse150 and TE-13 ESCC cells. We selected the most effective plasmid and confirmed the efficiency by western blot (Additional file 1: Figure S1B & S1C). As shown in Fig. 2, knockdown of S1PR1 decreased proliferation of kyse150 and TE-13 cells (Fig. 2a, c, Additional file 1: Figure S2A and S2C). Flow cytometric analysis of the cell cycle showed a decrease of cell number in S phase (Fig. 2b & Additional file 1: Figure S2B). Knockdown of S1PR1 elicited apoptosis of kyse150 and TE-13 cells (Fig. 2d, e, Additional file 1: Figure S2D and S2E). Consistently, western blot analysis showed decreased S1PR1, PCNA, BCL-XL, cyclinD1 and increased cleaved caspase-3 in S1PR1 knockdown cells, suggesting that silencing S1PR1 expression may induce cell death through apoptotic pathways (Fig. 2f & Additional file 1: Figure S2F).

### Overexpression of S1PR1 promoted proliferation and inhibited apoptosis of ESCC cells in vitro

**Fig. 3:**
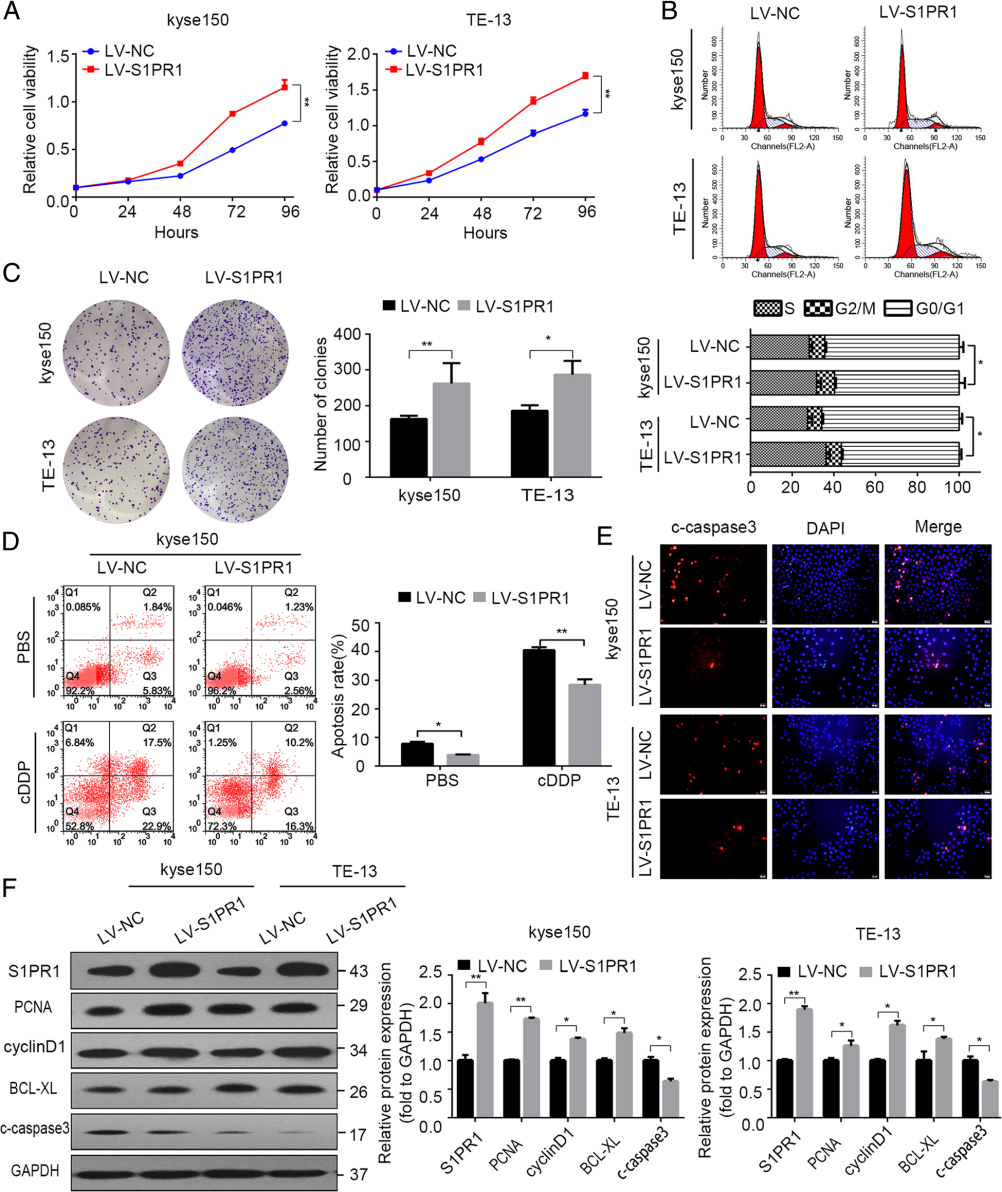
The effects of S1PR1 overexpression on cell proliferation, apoptosis of ESCC cells. **a** CCK-8 assay analysis of kyse150 and TE-13 cells infected with LV-NC or LV-S1PR1 respectively. **b** Flow cytometry analysis of cell cycle distribution proportion in kyse150 and TE-13 cells infected with LV-NC or LV-S1PR1 respectively. **c** Colony formation assay of kyse150 cells infected with LV-NC or LV-S1PR1 respectively treated with indicated concentrations of PBS and cDDP. **d** Flow cytometry analysis of apoptotic cells in kyse150 cells infected with LV-NC or LV-S1PR1 respectively treated with indicated concentrations of PBS and cDDP. **e** Immunofluorescent staining of c-caspase3 of kyse150 and TE-13 cells infected with LV-NC or LV-S1PR1 respectively. **f** Western blot analysis of S1PR1, PCNA, cyclinD1, Bcl-xL, c-caspase3 and GAPDH in kyse150 and TE-13 cells infected with LV-NC or LV-S1PR1 respectively

We next examined whether ectopic expression of S1PR1 could accelerate ESCC cell proliferation. Kyse150, TE-13 and TE-10 ESCC cells were infected with S1PR1 overexpressing lentivirus (Additional file 1: Figure S1D & S1E). As expected, overexpression of S1PR1 increased cell proliferation in both cells (Fig. 3a & Additional file 1: Figure S3A). S1PR1 overexpressing cells formed more clones than control cells (Fig. 3c). Furthermore, the flow cytometric analysis of the cell cycle showed that overexpression of S1PR1 resulted in an increase of cell number in the S phase (Fig. 3b & Additional file 1: Figure S3B). Overexpression of S1PR1 decreased apoptosis of kyse150 and TE-13 cells in ESCC (Fig. 3d, e, Additional file 1: Figure S3D and S3E). Additionally, increased S1PR1, PCNA, BCL-XL, cyclinD1 and decreased cleaved caspase-3 were observed in S1PR1 overexpression cells by western blot (Fig. 3f & Additional file 1: Figure S3F).

### S1PR1 interacted with STAT3 and activated the STAT3 pathway

**Fig. 4:**
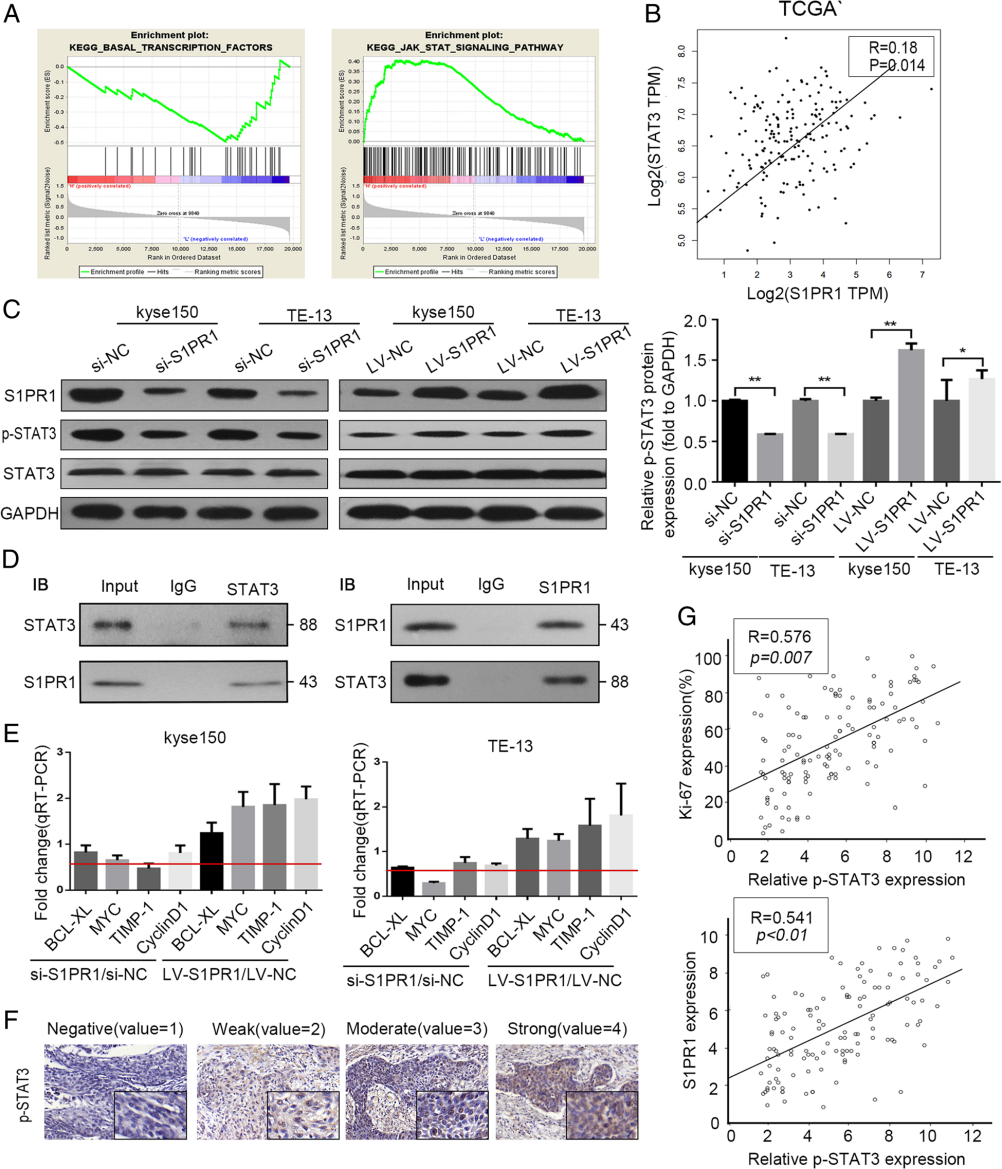
The interaction of S1PR1 with STAT3 and the expressions of STAT3-mediated transcription downstream targets. **a** GSEA plot indicating a significant correlation between the mRNA level of S1PR1 expression and the STAT3-activated signatures in TCGA ESCC specimen. **b** S1PR1 and STAT3 mRNA expressions from TCGA database of ESCC patients. **c** Western blot analysis of S1PR1, p-STAT3, total STAT3 and GAPDH in kyse150 and TE-13 cells with S1PR1 knockdown or overexpression. **d** Co-immunoprecipitation experiments were conducted on TE-13 cell lysates with anti-S1PR1and anti-STAT3, and then the precipitation was subsequently analyzed by western blotting with anti-STAT3 and anti-S1PR1. **e** QRT-PCR analysis of BCL-XL, myc, Timp-1, and cyclinD1 mRNA in kyse150 and TE-13 cells with S1PR1 knockdown or overexpression. **f** Representative images from immunohistochemically staining of p-STAT3 in ESCC patient tissues. **g** Spearman correlation analysis of the expression of p-STAT3 and expression of S1PR1 and Ki-67 in ESCC tissues. Statistical significance was determined by Student’s t test. *p < 0.05*

To further investigate how S1PR1 promote proliferation and inhibited apoptosis of ESCC cells, GSEA of TCGA database was used, and we found that S1PR1 was positively correlated with the STAT3 pathway in ESCC (Fig. 4a). We next explored the relationship between S1PR1 and STAT3 mRNA expression levels and found that expression of S1PR1 mRNA was positively correlated with that of STAT3 mRNA in the TCGA database (Fig. 4b). STAT3 signaling is initiated by phosphorylation of its tyrosine and serine which initiates complexation of phosphorylated STAT3 monomers (pSTAT3). It has been reported that S1PR1 is a key element triggering signal transducers and activators of transcription3 (STAT3). We examined the phosphorylation level of STAT3 in kyse150 and TE-13 cells with different S1PR1 expression by small interfering and overexpression vectors. In this study, phosphorylation of STAT3 was downregulated after transfected with S1PR1 small interfere RNA. While p-STAT3 was upregulated after transfected with S1PR1 expressing vectors(Fig. 4c). To address whether S1PR1 could bind to STAT3 with each other, we conducted immunoprecipitation with S1PR1 antibody or STAT3 antibody using cell lysates from TE-13 cell. As shown in Fig. 4d, we found that endogenous S1PR1 and STAT3 formed a complex in TE-13 cells, indicating that there was direct or indirect interaction between S1PR1 and STAT3. To further investigate the function of STAT3 affected by S1PR1, the expressions of STAT3 downstream target genes were examined by qRT-PCR. As shown in Fig. 4e, four STAT3 target genes (BCL-XL, myc, Timp-1, and cyclinD1) were decreased when S1PR1 was downregulated by small interfering RNA, and opposite results were observed after S1PR1 was upregulated (Fig. 4e). We next investigated p-STAT3 expression in the tissues chip of ESCC (Fig. 4f) and analyzed the relationship between p-STAT3 expression and S1PR1 expression or Ki-67. As shown in Fig. 4g, linear correlation analysis showed that p-STAT3 expression was inversely correlated with S1PR1 expression and Ki-67 in ESCC tissues.

### S1PR1 enhanced the proliferation and inhibited the apoptosis of ESCC cells via STAT3 signaling pathway

**Fig. 5:**
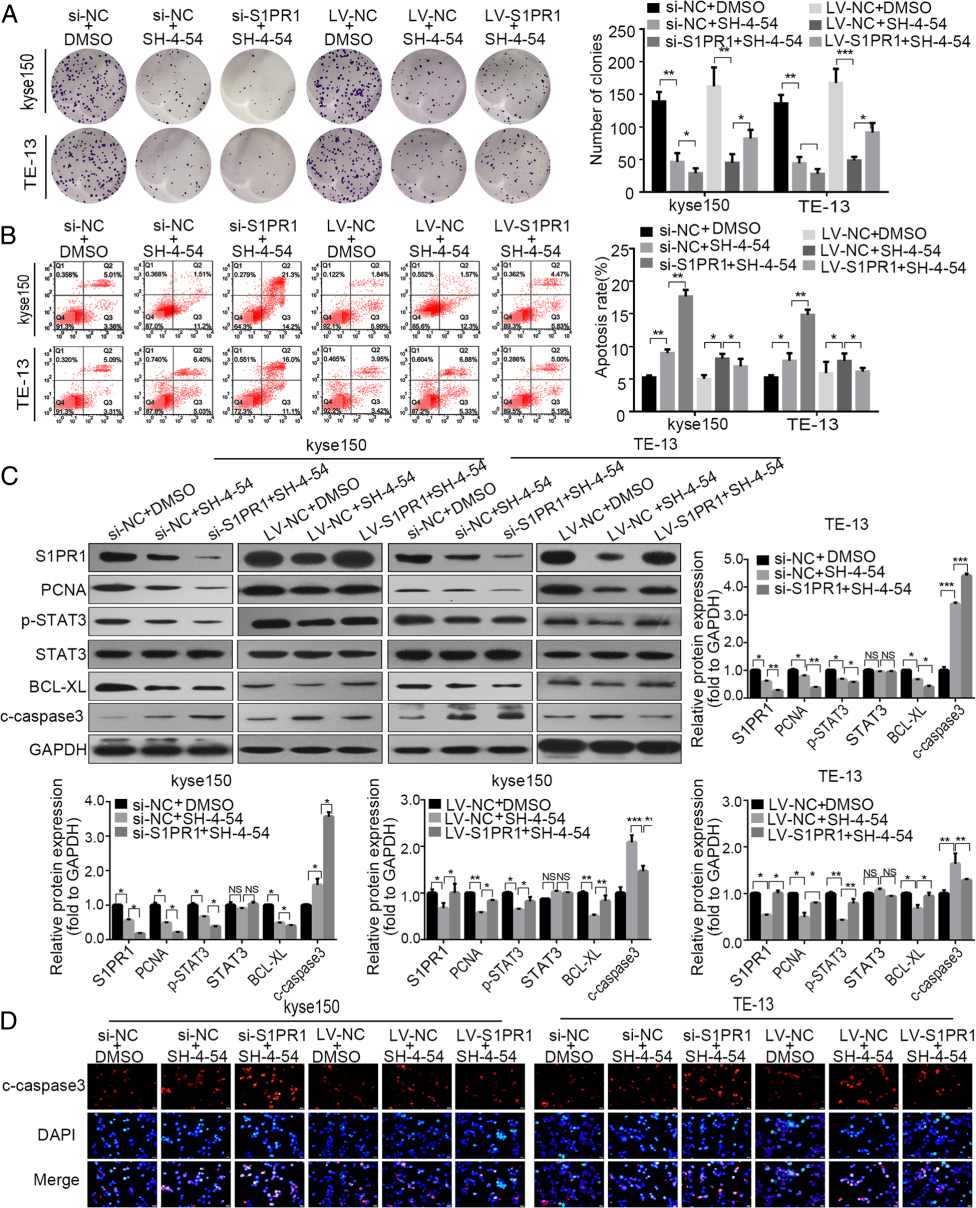
P-STAT3 inhibition inhibited proliferation and enhanced apoptosis of ESCC cells with S1PR1 knockdown or overexpression. **a** Colony formation assay of kyse150 and TE-13 cells with S1PR1 knockdown or overexpression treated with/without SH-4-54. **b** Flow cytometry analysis of apoptotic cells in kyse150 and TE-13 cells with S1PR1 knockdown or overexpression treated with/without SH-4-54. **c** Western blot analysis of PCNA, p-STAT3, STAT3, Bcl-xL, c-caspase3 and GAPDH in kyse150 and TE-13 cells with S1PR1 knockdown or overexpression treated with/without SH-4-54. **d** Immunofluorescent staining of c-caspase3 of kyse150 and TE-13 cells with S1PR1 knockdown or overexpression treated with/without SH-4-54

To investigate whether S1PR1 promotes ESCC proliferation through the STAT3 signaling pathway in ESCC cells, we inhibited p-STAT3 with SH-4-54 (phosphorylated STAT3 inhibitor as previously reported) [11]. We observed that kyse150 and TE-13 cells with high level of S1PR1 were more resistant to SH-4-54 than those with a low level of S1PR1 (Additional file 1: Figure S4A, B). As expected, inhibition of STAT3 attenuated colony formation ability and increased apoptosis in ESCC cells (Fig. 5a, b). Furthermore, we examined the expression of the p-STAT3 downstream proteins. Western blot demonstrated the downregulation of the proteins (p-STAT3, STAT3, PCNA, BCl-XL) and upregulation of cleaved-caspase-3 in kyse150 and TE-13 cells. Compared to single p-STAT3 inhibitor treatment, combined treatment with S1PR1 expression interfering and p-STAT3 inhibitor triggered lower colony formation ability and higher cell apoptosis rate (Fig. 5a and b). Moreover, p-STAT3, PCNA, Bcl-XL protein levels were significantly downregulated after combined treatment in kyse150 and TE-13 cells (Fig. 5c & d). To further validate these results, SH-4-54 was added into cells following overexpression of S1PR1, and the effects induced by S1PR1 overexpression were significantly inhibited. Besides, the upregulation of PCNA and p-STAT3, BCL-XL induced by LV-S1PR1 was reversed in the ESCC cell (Fig. 5c & d). Collectively, these results indicated that activation of STAT3 was necessary for S1PR1 in promoting tumor cell proliferation and inhibiting tumor cell apoptosis.

### S1PR1 promoted proliferation and inhibited apoptosis of ESCC in mouse xenograft models

**Fig. 6:**
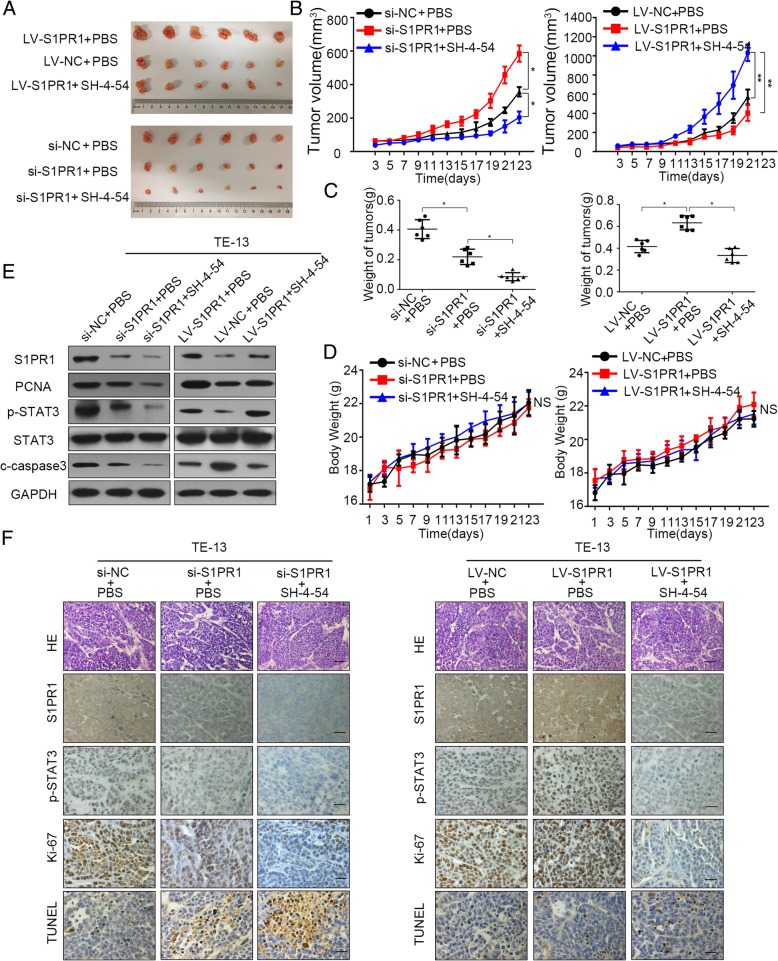
P-STAT3 inhibition inhibited proliferation and enhanced apoptosis with S1PR1 knockdown or overexpression in vitro. **a** Images of the xenograft tumors formed in BALB/c-Foxn1nu/Nju nude mice subcutaneously injected with TE-13 cells with S1PR1 knockdown or overexpression treated with/without SH-4-54. **b** TE-13 cells with S1PR1 knockdown or overexpression treated with/without SH-4-54. Tumor volumes of xenograft tumors are evaluated every 2 days. **c** Average weight of tumors derived from each group. d Body weight data for TE-13 xenograft mouse are evaluated every 2 days. **e** Western blot of S1PR1, PCNA, p-STAT3, STAT3 and c-caspase3 in xenografts from each group. Statistical significance was determined by Student’s t test. *p < 0.05.* f. H&E and immunostaining of S1PR1, p-STAT3, Ki-67 and TUNEL in xenografts from each group (scale bar, 100 μm). Statistical significance was determined by Student’s t test. *p < 0.05*

To further explore the co-treatment effects of S1PR1 and p-STAT3 inhibitor in vivo, we established TE-13 silencing and overexpressing xenograft models. Since the tumors grew up to 50 mm^3^, SH-4-54 was intraperitoneally injected into mice xenograft. Tumors derived from S1PR1-silenced cells grew slower than the control group. Compared to the group only with S1PR1 expression interference, combination of S1PR1 knockdown and SH-4-54 induced a significant reduction in tumor growth. Conversely, tumors formed by S1PR1-overexpressed TE-13 cells grew faster than those formed by LV-control cells, and this phenomenon was reversed by SH-4-54 (Fig. 6a, b and c). Additionally, the single drug or combination treatments had no significant effects on body weight of mice in different groups (Fig. 6d). Western blot and IHC analysis of xenografts revealed combining S1PR1 knockdown and STAT3 inhibitor remarkably decreased cell proliferation (Ki-67) and increased cell apoptosis (TUNEL), comparing with that in other groups. Besides, the STAT3 inhibitor reversed the effects of increased proliferation and decreased cell apoptosis bringing from S1PR1 overexpression ((Fig. 6e & f). Taken together, our in vivo experiments supplemented the in vitro observations.

**Fig. 7:**
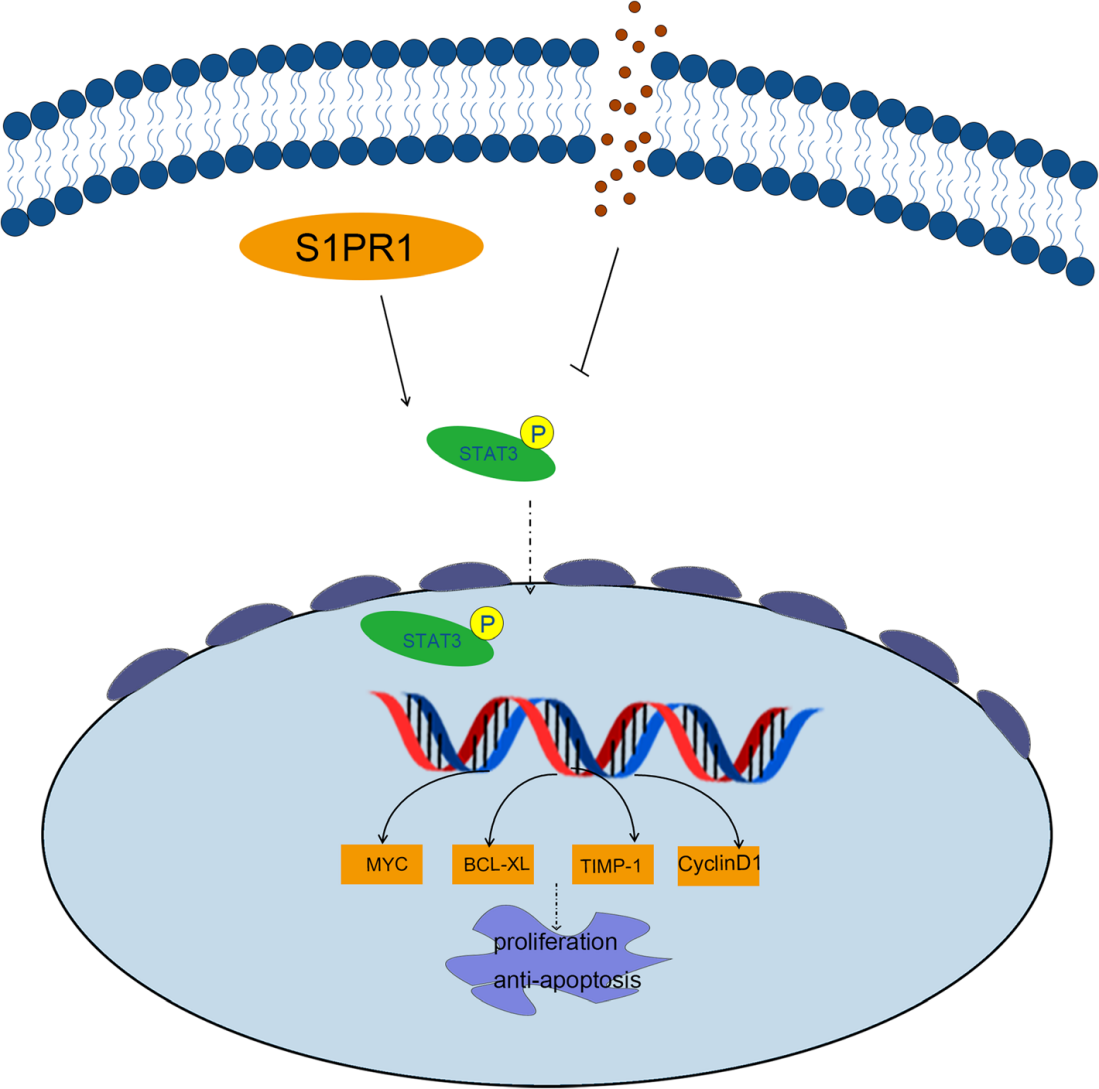
Systemic diagram of the mechanism of S1PR1/STAT3 signaling pathway. S1PR1 regulated the phosphorylation of STAT3 through directly binding to STAT3. Then phosphorylated STAT3 transferred into the nucleus to regulate the transcription of downstream target genes, leading to proliferation promotion and apoptosis inhibition of ESCC cells

## Discussion

Esophageal Squamous Cell Carcinoma harbored significant genetic heterogeneity. Due to the deficiency of efficient biomarkers, it was hard to discriminate ESCC patients with poor prognosis, who need more clinical surveillance, radiotherapy, chemotherapy, and target therapy, etc. Although lots of studies have been performed to identify prognostic markers for cancer-specific recurrence, progression, and death, there was no clinically verified predictor for ESCC patients until now [12–14]. Bioinformatics analysis of big data has revealed that aberrant expression of some factors, which act as potential biomarkers for cancer diagnosis or prognosis, may be critical in cancer development. Through searching the TCGA dataset, we found that S1PR1 was one of the most upregulated genes in ESCC patients with poor prognosis.

S1PR1 has been reported to be engaged in the regulation of cancer growth, proliferation, and apoptosis [15]. Previous studies have demonstrated that upregulation of S1PR1 was found in some solid human cancers, including breast cancer, gastric cancer and hepatocellular carcinoma (HCC) [5, 16–18]. And blocking the S1PR1 signaling pathway could inhibit tumor proliferation and induce apoptosis in multiple tumor cell lines (pancreatic cancer, renal cell carcinoma, and colorectal cancer) [19–21]. It has been reported that S1P/S1PR1 signaling pathway was involved in promoting cancer cell proliferation [22, 23]. Nevertheless, the S1PR1 could emit signals with the help of its downstream G protein partners without S1P [24]. A previous study detected the expression of S1PR1 in clinical ESCC tissues and confirmed that it was higher than adjacent normal tissues. However, the functions of S1PR1 in ESCC have been less explored. In our study, we discovered that S1PR1 was a predictor for poor prognosis in ESCC and its expression was positively correlated with proliferation ability of ESCC cells. Tissue homeostasis depends on the balance between cell proliferation and programmed cell death (apoptosis, autophagy, necroptosis, pyroptosis, etc.) [25, 26]. Numerous factors, such as p53, cellular inhibitor of apoptosis proteins (cIAPs), and radiation have been reported to regulate tumor apoptosis [27–29]. Also, it was illustrated that S1PR1 inhibited HCC apoptosis through activating MAPK signaling and reducing ROS level in AML cells [30, 31]. Consistent with previous studies, our results indicated that silencing S1PR1 expression induced apoptosis in kyse150 and TE-13 cells, while S1PR1 overexpression decreased the apoptosis rate of ESCC cells. Mechanistic studies revealed that TGF-β/smad3 could induce the upregulation of caspase3 via stimulating S1PR1, while S1PR1 could control BCL-2 level by modifying BCL-2a expression in melanoma cells [32, 33]. To better understand the molecular mechanism that S1PR1 regulates ESCC cancer cell apoptosis, we further examined the expression of proteins related to apoptosis. According to our observations, S1PR1 inhibited apoptosis of kyse150 and TE-13 cells by increasing the level of BCL-XL and preventing the cleavage of caspase-3.

With regards to the signaling pathways involved with the functions of S1PR1, Ras/Raf pathway, PI3K/Akt pathway, ERK pathway, and MAPK pathway have been focused recently [7, 9, 34]. Likewise, S1PR1 signaling inhibition treatment resulted in inhibition of cell growth in pancreatic cancer cells via STAT3 pathway [21]. STAT3, as a critical transcription factor, was highly phosphorylated in tumorigenesis and the level of phosphorylation was associated with worse prognosis in several cancers, including prostate cancer, HCC, and pancreatic cancer [35–37]. Previous studies confirmed that STAT3 acted directly on the proliferation and apoptosis in tumor cells [38, 39]. Activated by upstream signaling (cytokine family, activated STAT (PIAS) proteins and protein-tyrosine phosphatase (PTP) family), p-STAT3 dimerized, transferred into the nucleus then regulated the transcription of target genes [40, 41]. Structural studies revealed that the activation of STAT3 was initiated through the highly conserved SH2 domain, which comprised 140 amino acids and a distinct pTyr--recognition and pTyr-integration pocket [42]. The phosphorylation of the tyrosine residue (Y705) of STAT3 was firstly identified. Subsequently, STAT3 phosphorylation on S694, S727, K170, and K685 was also discovered to play important roles in oncogenic functions [43]. One study in MB49 tumor cells showed that the phosphorylation of C-terminal domain of S1PR1 could directly promoting consistent STAT3 activation [44]. Furthermore, it has been known that S1PR1 could be coupled to Gi, and then activates downstream kinases including tyrosine kinases (Src, Ras, JAK2) and serine/threonine kinases (GRKs and CamK) which both could induce STAT3 activation. Mechanically, it has been also reported that that S1PR1 induced JAK2/STAT3 activation through Y705 phosphorylation of STAT3, while induced mTOR/STAT3 activation through S727 phosphorylation of STAT3 [45]. Meanwhile, another study performed by Gough found Ras-mediated downstream transformation was significantly reduced when STAT3 mutated on its S727 residue [46]. These studies indicated that there might be existing different mechanisms underlying STAT3 activation in tumor cells. In our study, we observed that S1PR1, one of the GPCRs, could induce the phosphorylation of STAT3 and then increase the expressions of downstream genes and promote proliferation of ESCC cells (Fig. 7). Interestingly, S1PR1 was also one of the target genes of STAT3 and S1PR1/STAT3 formed a positive feedback loop, which might play important roles in the progression of pancreatic cancer [47].

## Conclusion

Our study demonstrated that high expression of S1PR1 contributed to the proliferation and survival of ESCC cells via activating STAT3 signaling pathway. Patients with high expression of S1PR1 had a poorer prognosis, indicating S1PR1 could be an effective prognostic predictor for ESCC patients.

## Additional file

Additional file 1: **Figure S1.** S1PR1 expression is significantly higher in the group with poor prognosis. **Figure S4.** ESCC cells with high level of S1PR1 were more resistant to SH-4-54. (PDF 1636 kb)

## Data Availability

The datasets supporting the conclusions of this article are included within the article.

## Abbreviations

AML: Acute myeloid leukemia
Bcl-2: B-cell lymphoma-2
BCL-XL: B-cell lymphoma-extra large
CCK-8: Cell Counting kit-8
Co-IP: Co-immunoprecipitation
DAB: Diaminobenzidine
DAPI: 4,6-diamino-2-phenyl indole
ERK: Extracellular regulated protein kinases
ESCC: Esophageal squamous cell carcinoma
FITC: Annexin V-fluorescein isothiocyanate
GPCR: G protein coupled receptors
GSEA: Gene set enrichment analysis
HCC: Hepatocellular carcinoma
IHC: Immunohistochemistry
MAPK: Mitogen-activated protein kinase
OS: Overall survival
PI3K: Phosphatidylinositol 3-kinase
PTP: Protein-tyrosine phosphatase
PVDF: Polyvinylidene fluoride
qRT-PCR: quantitative reverse-transcription polymerase chain reaction
S1P: Sphingosine-1- phosphate
Sphk: Sphingosine kinase
STAT3: signal transducing activator of transcription 3
TCGA: The Cancer Genome Atlas
Timp-1: Metallopeptidase inhibitor 1
TUNEL: Terminal deoxynucleotidyl transferase-mediated dUTP-biotin nick end labeling assay
