# Supplementary material for: S1PR1 promotes proliferation and inhibits apoptosis of esophageal squamous cell carcinoma through activating STAT3 pathway
Source: J Exp Clin Cancer Res. 2019 Aug 22;38:369. doi: 10.1186/s13046-019-1369-7 (PMC6706905; doi:10.1186/s13046-019-1369-7)
Supplement: Supplementary file 1 — Figure S1. S1PR1 expression is significantly higher in the group with poor prognosis. Figure S4. ESCC cells with high level of S1PR1 were more resistant to SH-4-54. (PDF 1636 kb) [file 13046_2019_1369_MOESM1_ESM.pdf]

Supplementary Material

Figure S1

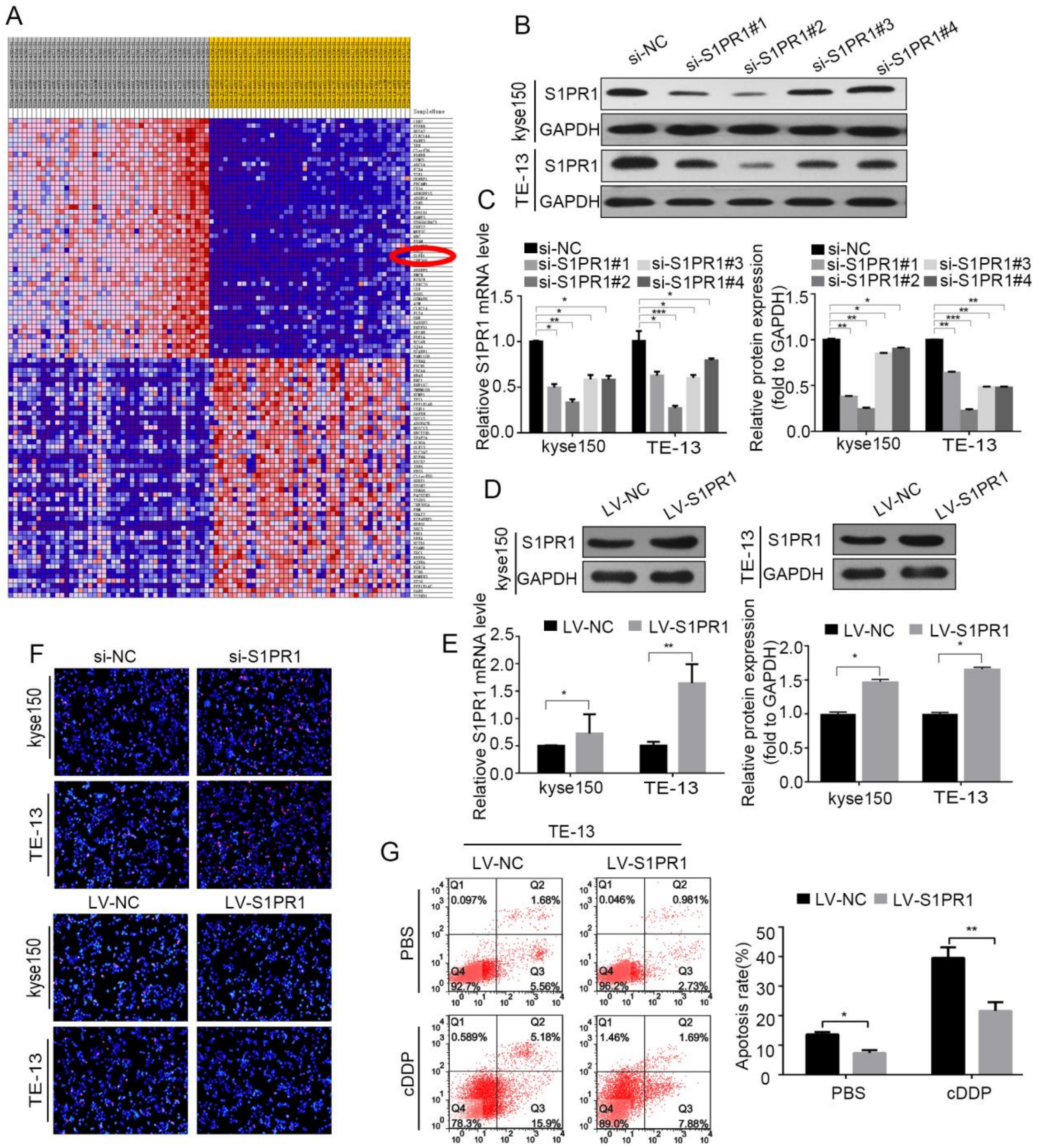

a. Heat map of gene levels of top upregulated expression genes and downregulated genes in TCGA patients. b.Western blot assay of S1PR1 protein in control and knockdown ESCC cells. c.QTR-PCR analysis of S1PR1 mRNA in control and knockdown ESCC cells. d. Western blot assay of S1PR1 protein in control and overexpression ESCC cells. e. QTR-PCR analysis of S1PR1 mRNA in control and overexpression ESCC cells. f. TUNEL staining of kyse150 and TE-13 cells transfected with si-NC or si-S1PR1 respectively. (upper). TUNEL staining of kyse150 and TE-13 cells infected with LV-NC or LV-S1PR1 respectively. g. Flow cytometry analysis of apoptotic cells in TE-13 cells infected with LV-NC or LV-S1PR1 respectively treated with indicated concentrations of PBS and cDDP.

Figure S2 The effects of S1PR1 downregulation on cell proliferation and apoptosis of ESCC cells.

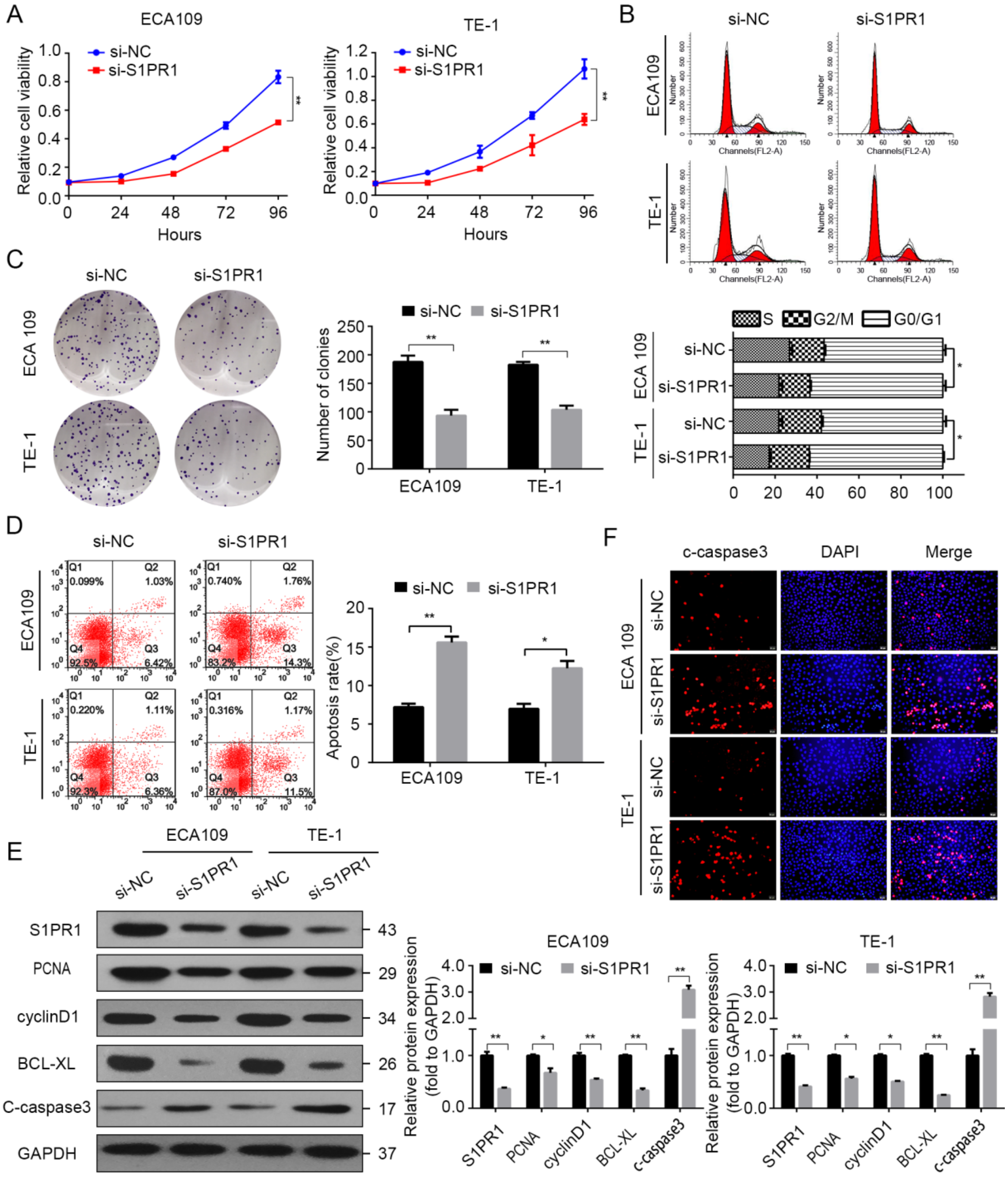

a. CCK-8 assay analysis of ECA109 and TE-1 cells transfected with si-NC or si-S1PR1 respectively. b. Flow cytometry analysis of cell cycle distribution proportion in ECA109 and TE-1 cells transfected with si-NC or si-S1PR1 respectively. Representative images and average percentages of cells are shown. c. Colony formation assay of ECA109 and TE-1 cells transfected with si-NC or si-S1PR1 respectively. d. Flow cytometry analysis of apoptotic cells in ECA109 and TE-1 cells transfected with si-NC or si-S1PR1 respectively. The sum of Annexin V positive population and PI positive population is exhibited. e. Western blot analysis of S1PR1, PCNA, cyclinD1, Bcl-xL, c-caspase3 and GAPDH in ECA109 and TE-1 cells transfected with si-NC or si-S1PR1 respectively. f. Immunofluorescent staining of c-caspase3 of ECA109 and TE-1 cells transfected with si-NC or si-S1PR1 respectively.

Figure S3 The effects of S1PR1 overexpression on cell proliferation, apoptosis of ESCC cells.

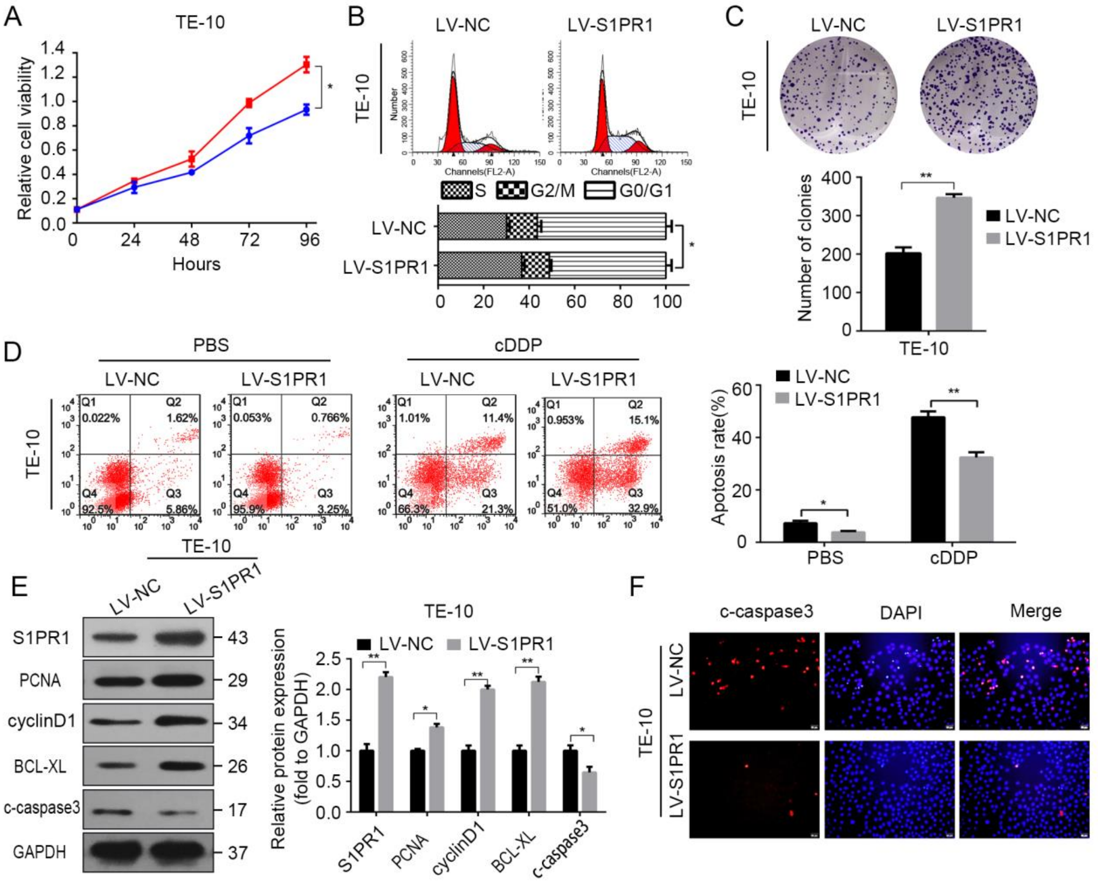

a. CCK-8 assay analysis of TE-10 cells infected with LV-NC or LV-S1PR1 respectively. b. Flow cytometry analysis of cell cycle distribution proportion in kyse150 and TE-13 cells infected with LV-NC or LV-S1PR1 respectively. c. Colony formation assay of TE-10 cells infected with LV-NC or LV-S1PR1 respectively. d. Flow cytometry analysis of apoptotic cells in TE-10 cells infected with LV-NC or LV-S1PR1 respectively treated with indicated concentrations of PBS and cDDP. e. Western blot analysis of S1PR1, PCNA, cyclinD1, Bcl-xL, c-caspase3 and GAPDH in TE-10 cells infected with LV-NC or LV-S1PR1 respectively. f. Immunofluorescent staining of c-caspase3 of TE-10 cells infected with LV-NC or LV-S1PR1 respectively.

Figure S4

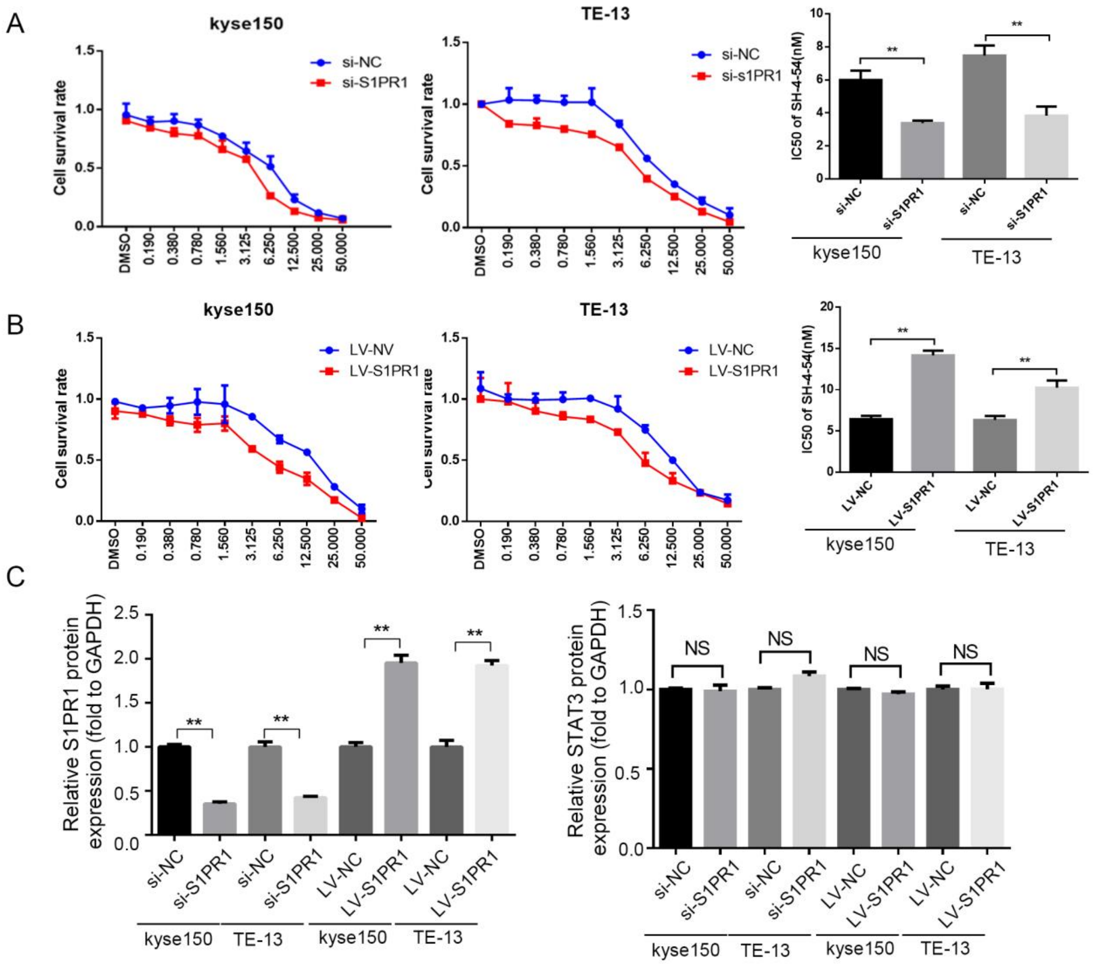

a. CCK-8 assay analysis of IC50 values of SH-4-54 in kyse150 and TE-13 cells transfected with si-NC or si-S1PR1 respectively. b. CCK-8 assay analysis of IC50 values of SH-4-54 in kyse150 and TE-13 cells infected with LV-NC or LV-S1PR1 respectively. c. Western blot analysis of S1PR1, total STAT3 in kyse150 and TE-13 cells with S1PR1 knockdown or overexpression.

Supplementary Table S1 Sequences of PCR primers used in this study

Gene	Primer sequence
S1PR1-F	CAGCAAATCGGACAATTCCT
S1PR1-R	GCCAGCGACCAAGTAAAGAG
BCL-XL-F	GAGCTGGTGGTTGACTTTCTC
BCL-XL-R	TCCATCTCCGATTCAGTCCCT
MYC-F	GGCTCCTGGCAAAAGGTCA
MYC-R	CTGCGTAGTTGTGCTGATGT
NOS2-F	TTCAGTATCACAACCTCAGCAAG
NOS2-R	TGGACCTGCAAGTTAAAATCCC
TIMP-1-F	CTTCTGCAATTCCGACCTCGT
TIMP-1-R	ACGCTGGTATAAGGTGGTCTG
CyclinD1-F	GCTGCGAAGTGGAAACCATC
CyclinD1-R	CCTCCTTCTGCACACATTTGAA
β-action-F	AGCGAGCATCCCCCAAAGTT
β-action-R	GGGCACCGAAGCTCATCATT
